# Comparative Genomic Analysis of Multidrug‐Resistant *Escherichia coli* Across Poultry–Human–Environmental Interfaces

**DOI:** 10.1002/mbo3.70406

**Published:** 2026-09-13

**Authors:** Md. Rokibul Hasan Shanto, A. S. M. Ashab Uddin, Md. Shakil Mahmud Supto, Nabil Jahan Mahim, Md. Matiar Rahman Howlader, Syed Sayeem Uddin Ahmed, Md Bashir Uddin

**Affiliations:** ^1^ Department of Medicine Sylhet Agricultural University Sylhet Bangladesh; ^2^ Bangladesh Livestock Research Institute (BLRI) Dhaka Bangladesh; ^3^ Department of Physiology Sylhet Agricultural University Sylhet Bangladesh; ^4^ Department of Epidemiology and Public Health Sylhet Agricultural University Sylhet Bangladesh

**Keywords:** antimicrobial resistance, Bangladesh, *E. coli*, one health, pangenome, poultry, ST457, virulence factors, whole‐genome sequencing

## Abstract

The emergence of multidrug‐resistant (MDR) *Escherichia coli* in poultry represents a critical One Health concern, particularly in developing countries. This study employed a comparative genomic approach to investigate the genomic characteristics, antimicrobial resistance (AMR) profiles, virulence determinants, of poultry‐derived MDR *E. coli* isolates from Bangladesh. Whole‐genome sequencing of three representative MDR isolates, identified with 83 globally diverse poultry, human, and environmental *E. coli* genomes. Pangenome analysis identified the characteristic open pangenome of *E. coli*, with core genes comprising only 4.6% of the combined dataset. Resistome analysis shown diverse AMR determinants, including *blaCTX‐M*, *blaTEM*, *sul*, *tet*, and *qnrS1*, associated with antibiotic inactivation and efflux mechanisms. Virulence profiling revealed diverse genes involved in adhesion (*fim*, *csg*), iron acquisition (*ent*, *fep*, *chu*), motility, and secretion systems, with core virulence genes exhibiting > 90% sequence identity, whereas accessory virulence genes were more variable. Plasmid analysis demonstrated heterogeneous replicon types, predominantly IncF and Col plasmids, indicating their role in horizontal gene transfer. Jaccard similarity indices revealed moderate to high genetic overlap with global strains (~0.63 for virulence genes and ~0.55 for AMR profiles), suggesting shared evolutionary backgrounds. Phylogenomic and MLST identified all Bangladeshi isolates as ST457, clustering within a globally distributed clonal complex linked to ST10 and ST131 lineages. These findings suggest that the three Bangladeshi poultry‐derived *E. coli* isolates are genetically related to globally circulating strains while harboring extensive resistance and virulence determinants, emphasizing poultry as an important reservoir of MDR pathogens and reinforcing the need for strengthened antimicrobial stewardship and genomic surveillance.

## Graphical abstract

Whole‐genome sequencing analysis of *Escherichia coli* isolates from broiler, layer and sonali chickens in Bangladesh shown extensive antimicrobial resistance, diverse virulence factors, plasmid‐mediated resistance, and a highly open pangenome. Comparative genomic analyses demonstrated close genetic relationships with global strains, highlighting the One Health significance of antimicrobial‐resistant *E. coli* transmission.

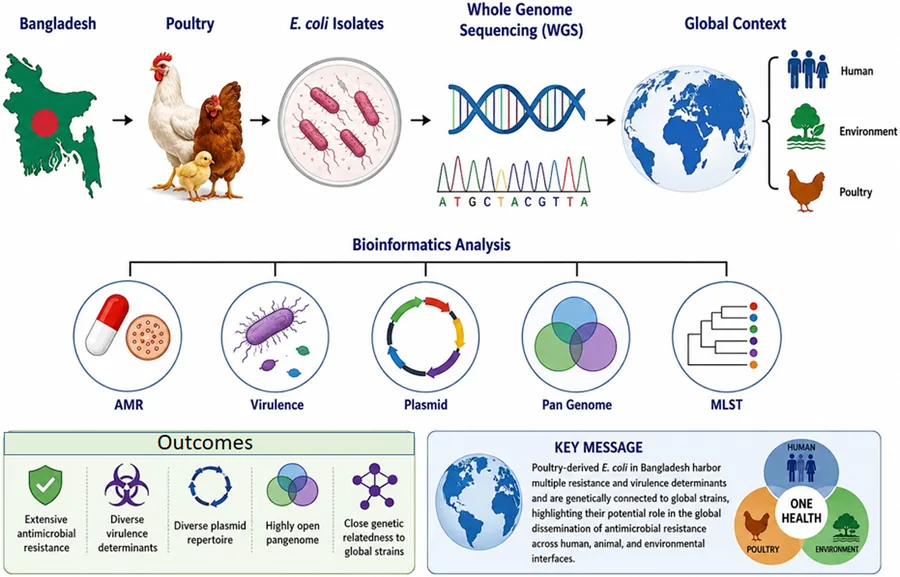

## 1 Introduction

Antimicrobial resistance (AMR) represents one of the most serious challenges that arises as a global health threat in the 21st century, significantly impairing all antimicrobials' efficacy both in human and veterinary medicine (Salam et al. 2023; Wah et al. 2023). Recent estimates showed that AMR resulted in approximately 1.27 million deaths worldwide in 2019, along with nearly 4.95 million associated deaths (Laxminarayan 2022). Besides its clinical impact, AMR is a substantial economic burden, and in the absence of effective mitigation measures, its global impact is projected to be USD 100 trillion by 2050 (Jain et al. 2024). In this respect, *E. coli* is a major concern due to its commensal and opportunistic pathogen status and its ability to acquire and disseminate antibiotic resistance determinants.

The poultry production systems have been highly regarded as potential sources of antimicrobial‐resistant *E. coli*, particularly in low and middle‐income countries that generally lack proper regulation on antimicrobial application (Abou‐jaoudeh et al. 2024; Hedman et al. 2020; Olaru et al. 2023). High stocking rates, intensive farming practices, and the continual use of antimicrobials as growth promoters and disease suppressors provide a sustained selective pressure promoting the emergence of multidrug‐resistant (MDR) strains (Prack McCormick et al. 2023). In Bangladesh, the poultry boom has exacerbated this scenario due to long‐standing availability of antibiotics over the counter without much regulation by the veterinary fraternity. Previously published studies reported a wide spectrum of antimicrobial resistance in poultry farms, including multidrug‐resistant *E. coli* with high prevalence rates in Bangladesh, where it is not uncommon to report resistance against widely used antibiotics such as tetracyclines, fluoroquinolones, and β‐lactams (Chowdhury et al. 2021; Ibrahim et al. 2023; Islam et al. 2023). These resistant strains could be transmitted to humans through direct contact, environmental contamination, and ingestion of contaminated poultry products, which highlights the importance of a One Health approach for AMR surveillance and management (Jothy et al. 2025; Singh et al. 2025).

*E. coli* is noteworthy for its genomic plasticity and inherent ability to readily adapt to a variety of ecological niches and selective pressures. Horizontal gene transfer (HGT) is a major mechanism in the acquisition and dissemination of antimicrobial resistance genes (ARGs), notably via mobile genetic elements such as plasmids, transposons, and integrons (Poirel et al. 2018; Shoaib et al. 2025; Tokuda and Shintani 2024). Widespread development of *E. coli* strains resistant to critically important antimicrobials, including extended‐spectrum 2‐lactam, fluoroquinolones, and polymyxins (e.g., colistin), is of concern worldwide (Dadashi et al. 2022; Paitan 2018). Extended‐spectrum β‐lactamase (ESBL) expressing *E. coli* and plasmid‐mediated colistin resistance determinants (the *mcr* genes) have severely limited treatment options, associated with decreased likelihood of successful treatment in human and veterinary medicine alike (Bitrus et al. 2018).

Whole‐genome sequencing (WGS) enables high‐resolution characterization of bacterial genomes, allowing the simultaneous identification of antimicrobial resistance genes, virulence factors, plasmid replicons, and sequence types, thereby providing a powerful tool for genomic surveillance and comparative analyses (Bertelli and Greub 2013; Zankari et al. 2013). Comparative pangenomic analyses have provided important insights into the genomic diversity, adaptation, and evolution of *E. coli*, particularly in relation to antimicrobial resistance and virulence (Dobrindt et al. 2010; Rasko et al. 2008). Genomic studies of poultry‐derived strains have identified numerous virulence determinants involved in adhesion, invasion, iron acquisition, and toxin production, many of which are characteristic of avian pathogenic *E. coli* (APEC) (Dziva et al. 2013; Mageiros et al. 2021). Moreover, the genetic similarity reported between certain APEC and extraintestinal pathogenic *E. coli* (ExPEC) strains highlights the potential public health relevance of poultry‐associated *E. coli* and supports the need for comparative genomic investigations (Moulin‐schouleur et al. 2007). Multilocus sequence typing (MLST) and phylogenomic analyses are widely used to investigate the population structure, clonal relatedness, and evolutionary relationships of *E. coli* lineages (Ewers et al. 2012; Matamoros et al. 2017). When combined with core genome phylogenetic analysis, these approaches provide valuable insights into the global distribution and evolutionary dynamics of bacterial lineages across animal, human, and environmental sources (Sintchenko and Holmes 2015).

Despite the rapid expansion of genomic datasets, comprehensive comparative genomic analyses of poultry‐derived MDR *E. coli* remain limited, particularly in low‐ and middle‐income countries. This limits our understanding of the genomic diversity and evolutionary characteristics of poultry‐associated *E. coli* circulating in Bangladesh. Therefore, this study employed a whole‐genome‐based comparative genomic approach to characterize multidrug‐resistant poultry‐derived *E. coli* isolates by investigating their antimicrobial resistance determinants, virulence gene repertoire, plasmid profiles, pangenome composition, sequence types, and phylogenetic relationships within a global genomic context. The findings provide genomic evidence that can support antimicrobial resistance surveillance, risk assessment, and future One Health strategies.

## 2 Materials and Methods

### 2.1 Ethical Consideration

This study involved non‐invasive collection of pooled poultry fecal samples from commercial farms and did not involve human participants or invasive animal procedures. Permission was obtained from poultry farm owners before sample collection. All sampling procedures and the use of animal‐derived biological materials were conducted in accordance with the ethical standards approved by the Animal Experimentation Ethics Committee of Bangladesh Livestock Research Institute (BLRI, approval #AEEC/BLRI/00022/2020). The study complied with applicable institutional guidelines for animal research.

### 2.2 Sample Collection and Isolation

A cross‐sectional study was conducted, during which samples were collected from the districts of Dhaka, Tangail, Mymensingh, and Sylhet. A total of 400 pooled fecal samples comprising 160 from broiler, 160 from layer, and 80 from Sonali poultry farms were included in the study. Each pooled sample was collected from five different locations or corners within a single farm, with one composite sample representing each farm. Pooled fecal samples were pre‐enriched in buffered peptone water (BPW), and incubated at 37°C for 18–24 h. Following enrichment, aliquots were streaked onto MacConkey agar and Eosin Methylene Blue (EMB) agar and incubated at 37°C for 18–24 h. Presumptive *E. coli* colonies were then selected based on colony morphology and subsequently confirmed by biochemical testing and PCR.

### 2.3 Molecular Identification

Genus‐level confirmation was performed using PCR targeting the 16S rRNA gene fragment of *E. coli*. Positive isolates were subjected to further PCR assays to differentiate common serovars through established primer sets (Table 1). The amplified PCR products were visualized by 1.5% agarose gel electrophoresis and further validated through sequencing.

**Table 1:** Primer set for *E. coli* identification.

Name of the primer	5′–3′	Base pair (bp)	Reference
*16S rRNA*	F‐GACCTCGGTTTAGTTCACAGA R‐CACACGCTGACGCTGACCA	585	Schippa et al. (2010)

### 2.4 Antimicrobial Susceptibility Testing

Confirmed *E. coli* isolates were then subjected to antimicrobial susceptibility testing (AST) using the Kirby–Bauer disc diffusion method on Mueller‐Hinton agar. A panel of twelve antimicrobials from nine different classes was used for AST. The panel included penicillin (ampicillin, 10 µg), beta‐lactamase inhibitors (amoxicillin‐clavulanate, 30 µg), carbapenem (imipenem, 10 µg), aminoglycosides (gentamicin, 10 µg), folate pathway inhibitors (sulfamethoxazole‐trimethoprim, 25 µg), cephalosporin/beta‐lactam antibiotics (cefoxitin, 10 µg; ceftriaxone, 30 µg; ceftazidime, 30 µg, cefepime, 30 µg), macrolides (azithromycin, 15 µg), quinolones/fluoroquinolones (ciprofloxacin, 5 µg), and tetracycline (tetracycline, 10 µg). Results were interpreted according to standardized guidelines, and MDR profiles will be identified (CLSI 2024).

### 2.5 Genome Sequencing and Assembly

Following PCR confirmation and antimicrobial susceptibility testing, three multidrug‐resistant *E. coli* isolates were purposively selected for whole‐genome sequencing. Selection was based on two criteria: (i) the isolates exhibited multidrug‐resistant phenotypes, and (ii) they represented the three major poultry production systems included in this study (broiler, layer, and Sonali), with one isolate selected from each production type. The isolates were selected to provide representative genomes for comparative genomic characterization and were not randomly chosen or selected based on phylogenetic relationships, which were determined only after genome sequencing. Sequencing libraries were prepared using the Nextera XT DNA Library Preparation Kit (Illumina Inc., USA), and whole‐genome sequencing was carried out on an Illumina NextSeq. 2000 platform to generate paired‐end reads. Approximately 8.2–9.8 million paired‐end read pairs (832.1–987.5 Mb of sequence data) were generated per isolate, resulting in an average genome coverage of approximately 300×. The raw reads were evaluated for quality using FastQC (v0.12.1) (Simon Andrews 2010), followed by trimming of low‐quality bases and adapter sequences using Fastp (v0.23.4) (Chen et al. 2018). High‐quality reads were assembled de novo using SPAdes (v4.0.0) with default parameters (Bankevich et al. 2012), and assembly quality was assessed using QUAST (v5.2.0) (Gurevich et al. 2013) based on standard metrics including genome size, number of contigs, and N50 values. Genome annotation was performed using the NCBI Prokaryotic Genome Annotation Pipeline (PGAP) which was used to predict protein‐coding genes, non‐coding genes, and pseudogenes. (Tatusova et al. 2016).

### 2.6 Genome Data Acquisition and Selection Parameters

In this study, a comparative genomic approach was employed, based on whole‐genome sequencing data of *E. coli*, with three poultry‐derived clinical isolates as the primary data and 83 publicly available genomes obtained from the National Center for Biotechnology Information (NCBI) GenBank (https://www.ncbi.nlm.nih.gov/genbank/) database to provide broad coverage of genomic diversity. The comparative dataset comprised poultry (*n* = 32), human (*n* = 31), and environmental (*n* = 20) isolates, yielding a total of 86 genomes for downstream analysis (Supplementary Table S1). Only high‐quality genome assemblies containing full metadata, such as accession number (GCA), strain name, host of origin, country of origin, and year of isolation, were included, and all sequences were obtained in both FASTA and GenBank formats to ensure uniformity and compatibility for subsequent analyses.

### 2.7 Genome Annotation

Functional annotation of all genomes in the dataset was performed using Prokka version 1.14.6 (Torsten Seemann 2014) with default parameters, a rapid and widely utilized tool for prokaryotic genome annotation. Each genome was processed individually, generating multiple output formats, including General Feature Format (GFF3), GenBank (GBK), protein sequences (FAA), and coding sequence nucleotide (FFN) files. Annotation involved the systematic identification of protein‐coding genes, ribosomal RNA (rRNA) genes, transfer RNA (tRNA) genes, and additional genomic features. The use of taxon‐specific databases ensured high specificity in gene detection. Annotated GFF3 files served as the primary input for subsequent pangenome analyses.

### 2.8 Detection of Antimicrobial Resistance Genes

Antimicrobial resistance (AMR) genes were identified from the assembled genomes of *E. coli* using ABRicate version 1.0.1 (Seemann 2017b) by screening nucleotide sequences against the ResFinder database (Bortolaia et al. 2020). Annotated genome assemblies were analyzed using default parameters to detect acquired resistance determinants associated with major antimicrobial classes, including beta‐lactams, tetracyclines, sulfonamides, and aminoglycosides. Detected genes were subsequently classified according to their corresponding antimicrobial categories, and resistance gene profiles were compiled across all isolates. For comparative analysis, a binary presence–absence matrix of AMR genes was constructed across all isolates, where detected genes were encoded as 1 and absence as 0. Pairwise similarity in AMR gene profiles between isolates was quantified using the Jaccard similarity index (Vargas and Juan 2016). Graphical visualization and statistical analyses were performed using R and Python.

### 2.9 Virulence Gene Profiling

Virulence‐associated genes were identified from the assembled genomes of *E. coli* using ABRicate (Seemann 2017b) by screening nucleotide sequences against the Virulence Factor Database (VFDB, version 6.0) (Liu et al. 2022). Genome assemblies were analyzed using default parameters to detect genes associated with key virulence functions, including adhesion, invasion, toxin production, iron acquisition, and immune evasion. Identified virulence genes were subsequently categorized based on their functional roles, and their distribution across isolates was compiled for comparative analysis. Comparative analysis of virulence gene profiles was performed using Jaccard similarity indices based on gene presence‐absence data. Visualization of virulence gene distribution and phylogenetic annotation was performed using iTOL (Letunic and Bork 2024), while additional graphical representations were generated using Python.

### 2.10 Plasmid Replicon Analysis

Plasmid replicon types were identified from the assembled genomes of *E. coli* by screening nucleotide sequences against the PlasmidFinder database (Carattoli et al. 2014). Genome assemblies were analyzed using default parameters to detect plasmid‐associated replicons, including common incompatibility groups such as IncF, IncI, and IncX. Similarity in plasmid replicon profiles between Bangladeshi isolates and global strains was assessed using Jaccard similarity indices. Visualization and graphical representation of plasmid structures and replicon distribution were performed using Proksee (Grant et al. 2023).

### 2.11 Pangenome Analysis

Pangenome analysis of *E. coli* genome datasets was done to detect common and variable components of genes. Roary (version 3.13.0) (Page et al. 2015) was used to analyze the annotated genome files (GFF3 format) to build the pangenome and produce gene presence‐absence matrices. Clustering of orthologous genes was performed by default, including the default minimum BLASTP protein identity threshold of 95%, with core genes defined as genes found in 99%–100% of isolates, accessory genes defined as genes present in multiple but not all isolates, and singleton genes defined as unique to an isolate. The pangenome outputs were subsequently visualized with Phandango (Hadfield et al. 2018), and further visualization of the three main poultry isolates was done with ImageGP 2 (Chen et al. 2024) to create a flower plot. In addition, Python was used to create a graphical representation of the genomic dataset to summarize the distribution of isolates and genomic features.

### 2.12 Phylogenetic Analysis and Visualization

Phylogenetic analysis was performed to determine the evolutionary relationships among the *E. coli* isolates included in this study. A gene presence–absence matrix generated from pangenome analysis using Roary was used to construct phylogenetic trees employing the FastTree algorithm with default parameters (Price et al. 2010). The resulting tree, based on core genome alignments, was exported in Newick format (Junier and Zdobnov 2010) for downstream analyses.

### 2.13 Multi‐Locus Sequence Typing (MLST) and Clonal Complex Analysis

Multilocus sequence typing (MLST) was performed on the assembled genomes of *E. coli* using the MLST tool (Seemann 2017a) to determine sequence types (STs) based on allelic profiles of conserved housekeeping genes following the Achtman scheme (Wirth et al. 2006). Genome assemblies were analyzed using default parameters, and sequence types were assigned according to corresponding allele combinations. Clonal complex (CC) analysis was subsequently inferred from the identified STs by grouping closely related sequence types based on shared allelic profiles using the eBURST algorithm (Francisco et al. 2009), where isolates sharing ≥ 6 of 7 alleles were assigned to the same CC. Isolates that could not be assigned to any clonal complex were categorized as unassigned. Visualization of sequence types and clonal complexes was performed using PHYLOViZ (Nascimento et al. 2017).

### 2.14 Jaccard Similarity Analysis

To assess the similarity of antimicrobial resistance gene profiles, virulence gene profiles, and plasmid replicon profiles among the analyzed isolates, binary presence–absence matrices were generated, where the presence of a feature was coded as 1 and its absence as 0. Pairwise similarities were calculated using the Jaccard similarity coefficient, which measures the proportion of shared features between isolates while excluding shared absences. For comparative analyses involving Bangladeshi isolates and isolates from other countries, mean Jaccard similarity values were calculated and used for downstream visualization and interpretation. All analyses were performed in Python using the Pandas, NumPy, Matplotlib, and Seaborn libraries. The Jaccard similarity analysis was intended to provide a descriptive assessment of gene‐content similarity among isolates. Consequently, confidence intervals, statistical significance testing, and adjustments for multiple comparisons were not included.

## 3 Results

### 3.1 Molecular Confirmation

Out of 400 pooled fecal samples, 280 were PCR positive. PCR amplification targeting the 16S rRNA gene produced the expected 585‐bp amplicon in all PCR‐positive isolates, confirming their identity as *E. coli* (Supplementary Figure S1). No amplification was observed in the negative control. Representative agarose gel electrophoresis demonstrated clear amplification of the target fragment, and the PCR products were subsequently confirmed by Sanger sequencing. Based on these molecular confirmation results, one representative isolate each from broiler, layer, and Sonali chickens was selected for whole‐genome sequencing (NGS).

### 3.2 Antimicrobial Drug Susceptibility of *E. coli* Isolates

Antimicrobial susceptibility testing demonstrated variable resistance patterns among the *E. coli* isolates. The highest resistance rates were observed against ampicillin (97.86%; 95% CI: 95.40–99.01), tetracycline (97.50%; 95% CI: 94.93–98.78), and trimethoprim–sulfamethoxazole (91.79%; 95% CI: 87.98–94.46). and ciprofloxacin (68.93%; 95% CI: 63.28–74.06). Moderate resistance frequencies were observed for cefoxitin, cefepime, whereas azithromycin and imipenem (96.43%; 95% CI: 93.55–98.05), showed the highest susceptibility (Supplementary Table S2).

### 3.3 Genome Assembly and General Features

The assembled genomes showed variability in quality and size among the isolates. Genome sizes ranged from 4.4 to 5.4 Mb, with GC contents between 50% and 51%. The number of contigs varied widely, from 246 to 6222, reflecting differences in assembly continuity. The N50 values ranged from 1.4 to 35.6 kb. Among the isolates, ARAC‐SAU‐CH‐3688 demonstrated the highest assembly quality with the lowest number of contigs and the highest N50 value, whereas ARAC‐SAU‐CH‐3762 exhibited a highly fragmented assembly. The total number of predicted genes ranged from 4309 to 10,175. Detailed genome features of the isolates are summarized in Table 2.

**Table 2:** Metadata of *E. coli* isolates included in this genomic study.

Strain ID	*E. coli*_SAU_Broiler	*E. coli*_SAU_Layer	*E. coli*_SAU_Broiler
Species	*E. coli*	*E. coli*	*E. coli*
Sample	Chicken feces (Broiler)	Chicken feces (Layer)	Chicken feces (Sonali)
BioSample accession no.	SAMN50861382	SAMN51409036	SAMN51409148
GenBank accession no.	GCA_052492195.1	GCA_053051155.1	GCA_052906445.1
RefSeq accession no	GCF_052492195.1	—	GCF_052906445.1
WGS project	JBQVYW01	JBRNVW01	JBRECO01
CC	—	—	—
ST	—	—	—
Genome size (bp)	5,100,000	5,400,000	4,400,000
GC content (%)	50	50	51
No. of contigs	385	6222	246
N50 (bp)	22,600	1400	35,600
No. of paired‐end read pairs	9,200,972	9,772,147	8,234,418
Coverage (×)	300	300	300
Total annotated genes (PGAP)	5111	10,175	4309

### 3.4 Distribution and Comparative Analysis of Antimicrobial Resistance (AMR) Genes

Comprehensive analysis of antimicrobial resistance (AMR) genes in the Bangladeshi *E. coli* isolates revealed the presence of multiple resistance determinants within each genome, indicating diverse resistance profiles (Table 3).

**Table 3:** Antimicrobial resistance genes and predicted resistance profiles of Bangladeshi *E. coli* isolates.

Isolate	Gene	Resistance
*E. coli*_SAU_Broiler	*CmlA7_1, FloR_4, aph(3″)‐Ib_5, aph(6)‐Id_1, blaCTX‐M‐27_1, dfrA14_5, sul2_2, tet(A)_6*	Doxycycline, tetracycline, chloramphenicol, florfenicol, streptomycin, amoxicillin, ampicillin, aztreonam, cefepime, cefotaxime, ceftazidime, ceftriaxone, piperacillin, ticarcillin, sulfamethoxazole, trimethoprim
*E. coli*_SAU_Layer	*CmlA7_1, FloR_4, aac(6′)‐Ib‐cr_1, aph(3″)‐Ib_5, aph(6)‐Id_1, blaCTX‐M‐15_1, blaOXA‐1_1, blaTEM‐1B_1, dfrA12_8, dfrA14_5, qnrS1_1, sul2_2, sul3_2, tet(A)_6, tet(M)_8*	Amikacin, amoxicillin, amoxicillin+clavulanic acid, ampicillin, aztreonam, cefepime, cefotaxime, ceftazidime, ceftriaxone, cephalothin, chloramphenicol, ciprofloxacin, dibekacin, doxycycline, florfenicol, fluoroquinolone, minocycline, netilmicin, piperacillin, piperacillin+tazobactam, sisomicin, streptomycin, sulfamethoxazole, tetracycline, ticarcillin, tobramycin, trimethoprim
*E. coli*_SAU_Sonali	*blaCTX‐M‐15_1, blaTEM‐1B_1, dfrA14_5, mph(A)_2, qnrS1_1, sul3_2*	Ciprofloxacin, azithromycin, erythromycin, spiramycin, telithromycin, amoxicillin, ampicillin, cephalothin, piperacillin, ticarcillin, aztreonam, cefepime, cefotaxime, ceftazidime, ceftriaxone, sulfamethoxazole, trimethoprim

Notably, isolate *E. coli*_SAU_Layer harbored a broad spectrum of AMR genes, including *blaCTX‐M‐15*, *blaOXA‐1*, *blaTEM‐1B*, *qnrS1*, *sul2/sul3*, *tet(A/M)*, and *aac(6′)‐Ib‐cr*, reflecting a comparatively higher resistance burden. These genes are associated with multiple resistance mechanisms, including antibiotic inactivation, target protection and alteration, and efflux‐mediated resistance, contributing to resistance against a wide range of antimicrobial classes. Similarly, isolates *E. coli*_SAU_Broiler and *E. coli*_SAU_Sonali contained key resistance genes such as *blaCTX‐M* variants, sul genes, *tet(A)*, *mph(A)*, and *dfrA*, which are linked to mechanisms including antibiotic inactivation, target alteration, and drug efflux, conferring resistance to beta‐lactams, tetracyclines, sulfonamides, aminoglycosides, and macrolides. The variation in both the number and type of AMR genes among these isolates highlights heterogeneity in resistance profiles and indicates the presence of multidrug resistance‐associated determinants. Analysis of the complete dataset further demonstrated extensive diversity in the distribution of AMR genes across *E. coli* isolates. The Sankey diagram (Figure 1A) illustrated the relationships among resistance genes, their associated mechanisms, and antimicrobial classes, with antibiotic inactivation and efflux‐related pathways prominently represented.

Figure 1Comparative analysis of antimicrobial resistance (AMR) profiles in *E. coli*. (A) Sankey diagram illustrating the relationships between resistance genes, associated mechanisms, and antimicrobial classes. (B) Heatmap showing the presence–absence patterns of AMR genes across isolates, with hierarchical clustering and metadata annotation; isolates highlighted with red boxes indicate the Bangladeshi strains included in this study. (C) Bar plot representing the average AMR similarity between Bangladesh isolates and different countries. (D) Bar plot displaying the top 10 most similar global strains to Bangladesh isolates based on Jaccard similarity.

**Figure:**
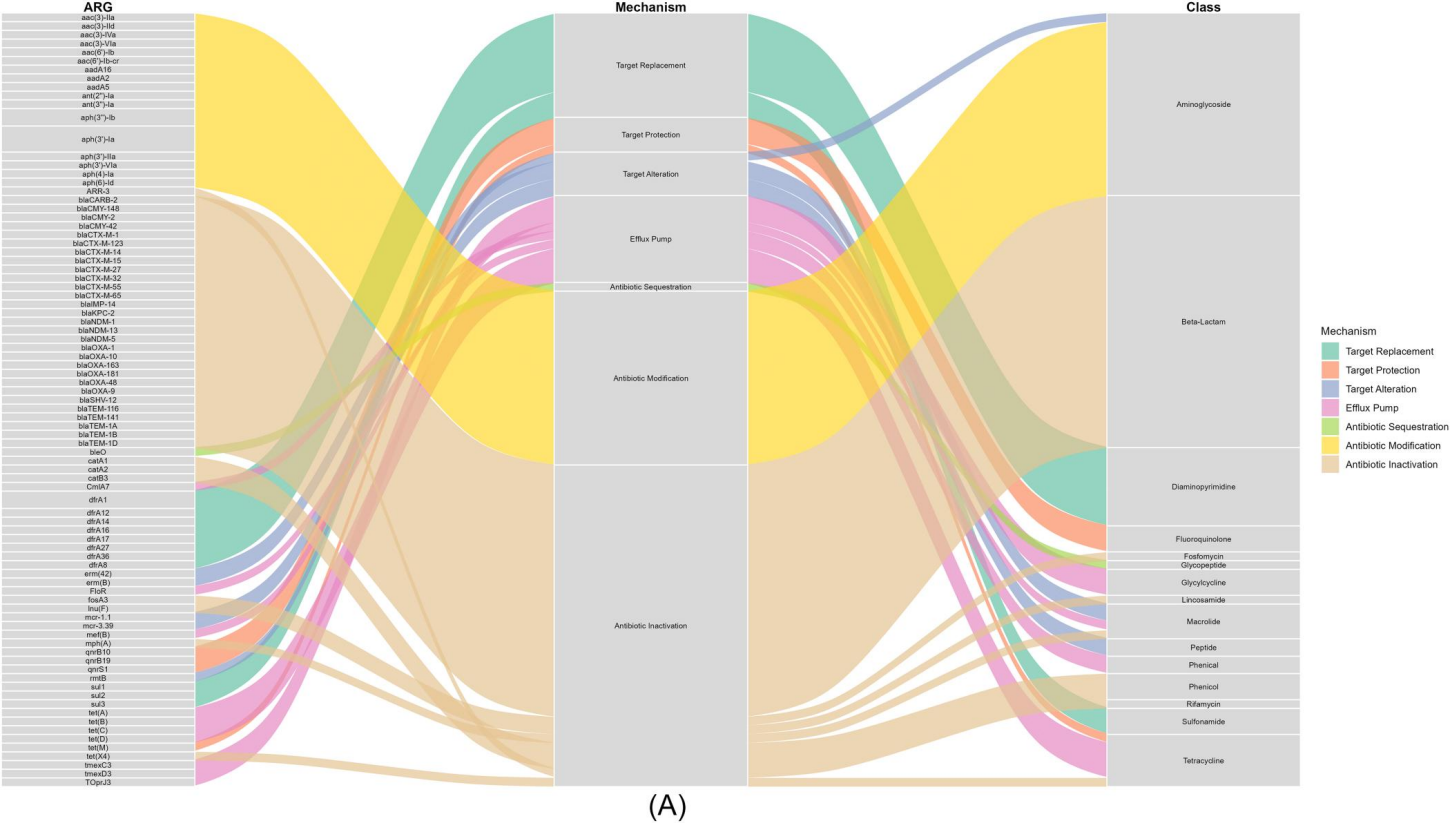

**Figure:**
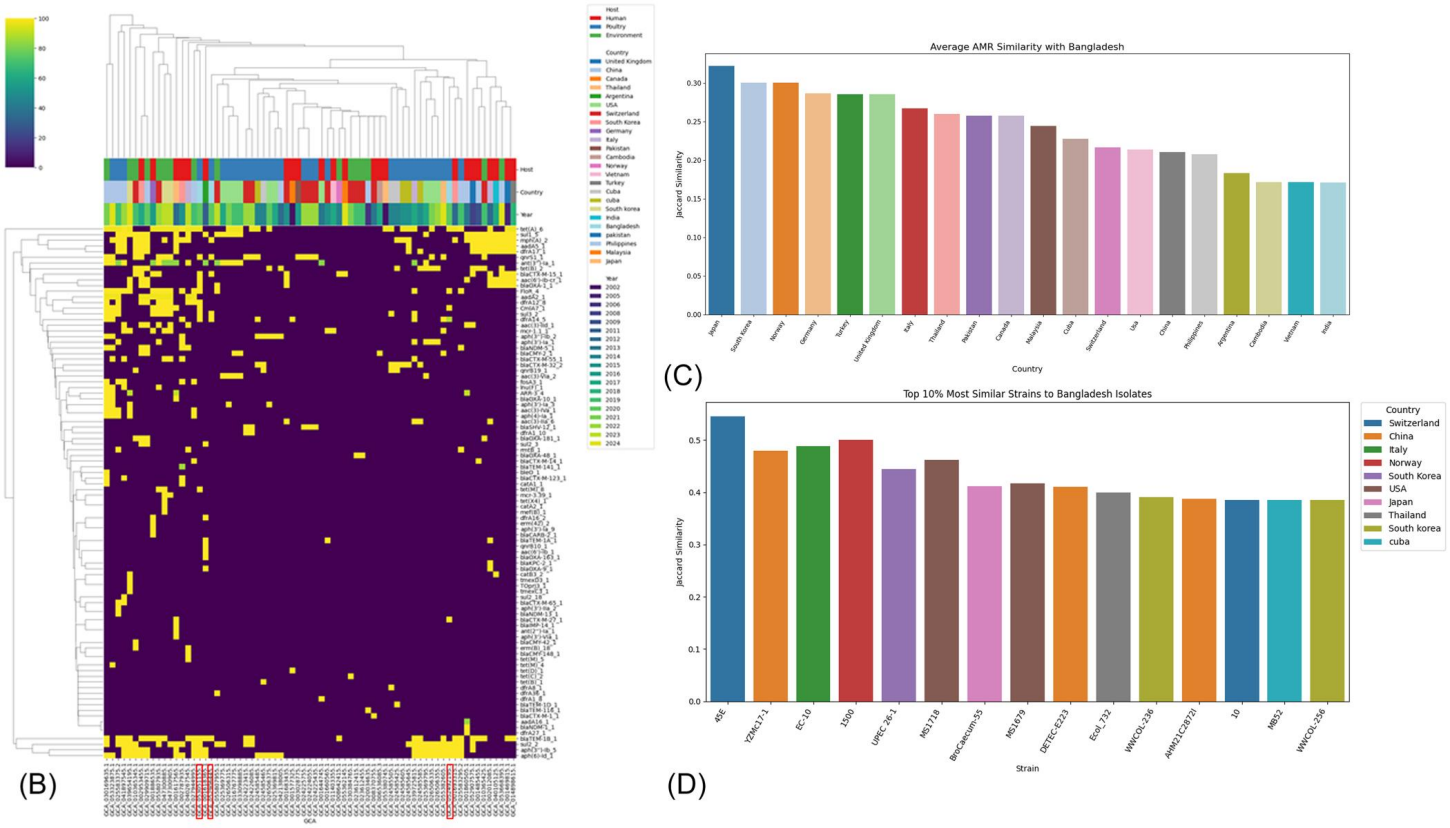

In parallel, heatmap‐based clustering (Figure 1B) revealed substantial variability in AMR gene presence across isolates, with clustering patterns reflecting differences in host, geographic origin, and year of isolation. Frequently detected genes across the dataset included *blaCTX‐M* variants, *blaTEM*, *sul1/sul2*, *tet(A/B)*, *aadA*, and *mph(A)*, indicating widespread resistance across major antimicrobial groups. Comparative analysis positioned the Bangladesh isolates within a broader global context.

Country‐level comparison (Figure 1C) showed that the highest average AMR similarity was observed with isolates from Japan (~0.32), followed by South Korea and Norway (~0.30), whereas lower similarity values were observed for countries such as Argentina, Cambodia, Vietnam, and India (~0.17–0.19). At the strain level (Figure 1D), the highest Jaccard similarity was observed for strain 45E (0.55, Switzerland), followed by strains 1500 (0.50, Norway), EC10 (0.49, Italy), and YZMC17‐1 (0.48, China), with additional related strains including MS1718 (0.46, USA) and UPEC26‐1 (0.44, South Korea). These findings highlight both shared and distinct resistance patterns between Bangladesh isolates and global *E. coli* populations.

### 3.5 Virulence Gene Diversity, Functional Categories, and Comparative Genomics

Virulence profiling of the three Bangladeshi *E. coli* isolates revealed a diverse distribution of virulence‐associated genes across multiple functional categories, including adherence, invasion, motility, effector delivery systems, and nutritional/metabolic factors (Table 4).

**Table 4:** Functional categorization and distribution of virulence‐associated genes identified in Bangladeshi *E. coli* isolates.

Isolate	Functional category	Genes
*E. coli_*SAU_Broiler	Adherence	*cfaA, cfaB, cfaC, cgsD, cgsE, cgsF, cgsG, csgA, csgB, csgC, fdeC, fimA, fimB, fimC, fimD, fimE, fimF, fimG, fimH, fimI, papB, papI, sfaX, yagV/ecpE, yagW/ecpD, yagX/ecpC, yagY/ecpB, yagZ/ecpA, ykgK/ecpR*
	Antimicrobial activity/competitive advantage	*acrB*
	Effector delivery system	*clpV/tssH, espL1, espR1, espX4, espY2, espY4, gspC, gspD, gspE, gspF, gspG, gspH, gspI, gspJ, gspK, gspL, gspM, hcp2/tssD2, tssA, tssJ, tssM*
	Immune modulation	*gndA*
	Invasion	*aslA, ibeB, ibeC, kpsC, kpsD, kpsE, kpsF, kpsM, kpsS, kpsU, ompA*
	Motility	*cheB, cheW, cheY, cheZ, flgC, flgG, flgH, flgI, flhA, flhC, fliE, fliG, fliI, fliM, fliN, fliP, fliQ, motA, motB*
	Nutritional/metabolic factor	*allB, chuA, chuS, chuT, chuU, chuV, chuW, chuX, chuY, entA, entB, entC, entD, entE, entF, entS, fepA, fepB, fepC, fepD, fepE, fepG, fes, iucA, iucB, iucC, iucD, iutA*
	Regulation	*fur, phoP, pmrA, rcsB, rpoS*
*E. coli_*SAU_Layer	Adherence	*cfaA, cfaB, cfaC, cgsE, cgsF, csgB, fimA, fimB, fimC, fimD, fimE, fimF, fimG, fimH, fimI, papB, papI, yagZ/ecpA*
	Effector delivery system	*clpV/tssH, espX1, espX5, espY2, espY4, gspC, gspD, gspE, gspF, gspG, gspI, gspJ, gspK, gspM, hcp1/tssD1, hcp2/tssD2, tssA, tssB, tssC, tssG, tssJ, tssK, tssL, tssM*
	Invasion	*kpsS, ompA*
	Motility	*cheW, cheY, fliE, fliG, fliQ*
	Nutritional/metabolic factor	*chuA, chuS, chuT, chuU, chuW, chuX, chuY, entB, entD, fyuA/psn, iroB, iroN, irp1, irp2, shuV, ybtA, ybtE, ybtP, ybtQ, ybtS, ybtT, ybtU, ybtX*
	Regulation	*fur, phoP, rcsB, rpoS*
*E. coli_*SAU_Sonali	Adherence	*cfaA, cfaB, cfaC, cfaD/cfaE, cgsD, cgsE, cgsF, cgsG, csgA, csgB, csgC, fimA, fimB, fimC, fimD, fimE, fimF, fimG, fimH, fimI*
	Antimicrobial activity/competitive advantage	*acrB*
	Effector delivery system	*espL1, espR1, espX1, espX4, espX5, gspC, gspD, gspE, gspF, gspG, gspH, gspI, gspJ, gspK, gspL, gspM*
	Immune modulation	*gndA, gtrA*
	Invasion	*ibeB, ibeC, ompA*
	Motility	*cheB, cheW, cheY, cheZ, flgC, flgG, flgH, flgI, flhA, flhC, fliE, fliG, fliI, fliM, fliN, fliP, fliQ, motA, motB*
	Nutritional/metabolic factor	*allB, entA, entB, entC, entE, entF, entS, fepA, fepB, fepC, fepD, fepE, fepG, fes*
	Regulation	*fur, phoP, pmrA, rcsB, rpoS*

**Figure 2:**
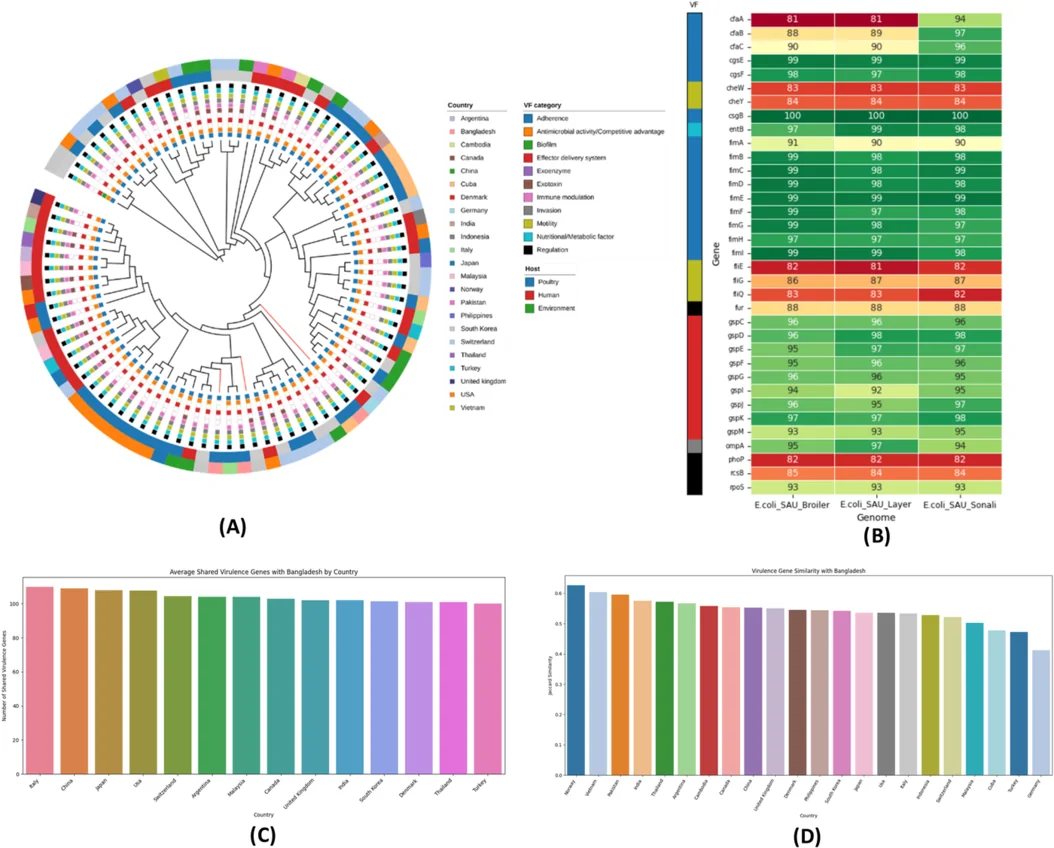
Virulence gene profiling and comparative analysis of *E. coli* isolates. (A) Combined visualization showing a circular phylogenetic tree of global isolates based on virulence gene profiles, where Bangladeshi isolates are highlighted in red branches, alongside a heatmap representing the presence and sequence identity of selected virulence genes across the three study isolates. (B) Bar plot illustrating the average number of shared virulence genes between Bangladesh isolates and isolates from different countries. (C) Bar plot showing Jaccard similarity of virulence gene profiles between Bangladesh isolates and global *E. coli* strains. (D) Bar plot showing Jaccard similarity of virulence gene profiles between Bangladesh isolates and global *E. coli* strains.

All three isolates possessed a conserved set of adhesion‐related genes, particularly the fimbrial cluster (*fimA–fimI*) and curli‐associated genes (*csgA–csgC, cgsD–cgsG*), indicating strong colonization potential. The broiler isolate exhibited the highest number of virulence genes, including an extensive repertoire of effector delivery system components such as type II and type VI secretion system genes (*gsp* and *tss* clusters), as well as invasion‐associated genes (*ibeB, ibeC, kps* gene cluster, *ompA*). In contrast, the layer isolate showed a comparatively reduced virulence gene repertoire, primarily consisting of adhesion, secretion system, and iron acquisition‐related genes. The Sonali isolate displayed an intermediate profile, with additional immune modulation genes (*gndA*, *gtrA*) and antimicrobial competitive advantage gene *acrB*. Across all isolates, motility‐related genes (*che, flg, fli*, mot families), iron acquisition systems (*ent, fep, chu* clusters), and regulatory genes (*fur, phoP, pmrA, rcsB, rpoS*) were consistently present, highlighting conserved mechanisms of host adaptation and survival. Phylogenetic analysis based on virulence gene profiles demonstrated that the Bangladeshi isolates were distributed among globally diverse *E. coli* lineages rather than forming a distinct cluster (Figure 2).

The isolates, highlighted within the phylogenetic tree, showed close relationships with strains from multiple countries, suggesting shared evolutionary backgrounds and potential dissemination of virulence determinants across regions. In the same figure (Figure 2B), the heatmap representing selected virulence genes across the three isolates showed high sequence identity (mostly >90%–100%) for core virulence determinants, particularly those associated with adhesion (*fim* genes), secretion systems (*gsp* genes), and iron acquisition. However, variation was observed in specific genes such as *che, fli*, and *phoP*, indicating minor differences in virulence gene composition among the isolates. Comparative analysis further positioned the Bangladesh isolates within a broader global context. The average number of shared virulence genes was highest with isolates from Italy (~110 genes), China (~109), Japan (~108), and the United States (~ 108) (Figure 2C), indicating substantial overlap in virulence gene content. Consistent with this, Jaccard similarity analysis showed the highest similarity with isolates from Norway (~0.63), followed by Vietnam (~0.60), Pakistan (~0.59), and India (~0.57), whereas lower similarity values were observed for Germany (~0.41), Turkey (~0.47), and Cuba (~0.48) (Figure 2D). These findings suggest that while a conserved core set of virulence genes is widely distributed, regional variation exists in gene composition and abundance, reflecting differences in ecological adaptation and evolutionary dynamics among global *E. coli* populations.

### 3.6 Plasmid Diversity and Structural Features in *Escherichia coli*

**Figure 3:**
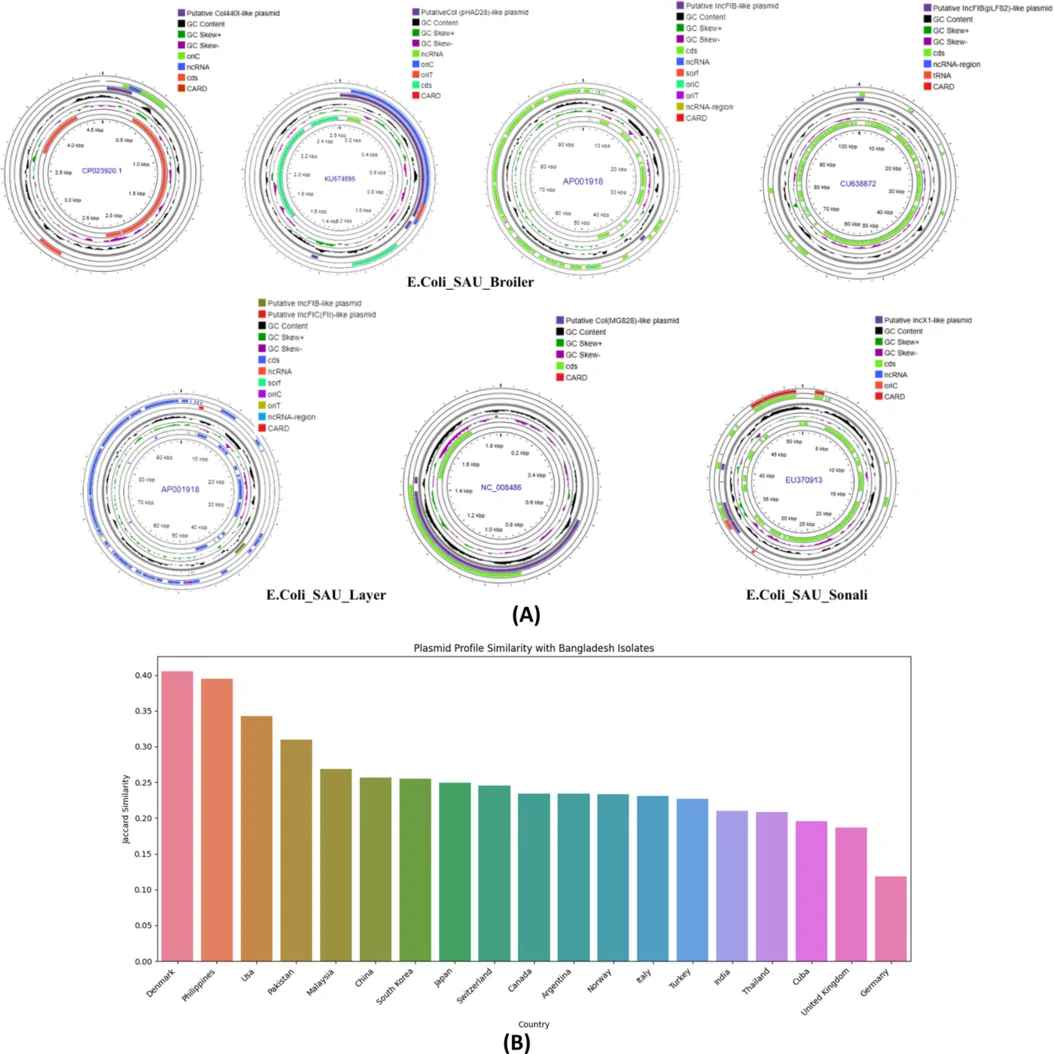
Plasmid profiling and comparative analysis of *E. coli* isolates. (A) Circular maps of plasmid sequences from the three Bangladeshi isolates (*E. Coli_*SAU_Broiler, Layer, and Sonali), showing genomic features including coding sequences (CDS), GC content, GC skew, non‐coding RNA (ncRNA), and origin‐associated regions (oriC/oriT). Different plasmid replicon types are indicated within each map. (B) Bar plot illustrating Jaccard similarity of plasmid profiles between Bangladesh isolates and global *E. coli* strains based on presence–absence of plasmid types.

Plasmid profiling of the three Bangladeshi *E. coli* isolates revealed distinct yet partially overlapping plasmid compositions and structural features (Table 5, Figure 3).

**Table 5:** Distribution of plasmid replicon types among Bangladeshi *E. coli* isolates.

Isolate	Plasmid types
*E. coli*_SAU_Broiler	Col(pHAD28), Col440I, IncFIB, IncFIB(pLF82),
*E. coli*_SAU_Layer	Col(MG828), IncFIB, IncFIC(FII)
*E. coli*_SAU_Sonali	IncX1

The *E. coli*_SAU_Broiler isolate exhibited the most complex plasmid architecture, harboring multiple replicon types including Col(pHAD28), Col440I, and IncF‐family plasmids such as IncFIB and IncFIB(pLF82), with well‐distributed coding sequences (CDS), clear GC content and GC skew patterns, and the presence of ncRNA elements and origin‐associated regions (oriC/oriT), while no CARD‐associated resistance regions were detected. In contrast, the *E. coli*_SAU_Layer isolate contained a comparatively moderate plasmid repertoire consisting of Col (MG828) and IncF‐type plasmids (IncFIB and IncFIC(FII)), where CDS regions, GC content, and oriC/oriT features were evident, although ncRNA elements were less prominent, and no resistance‐associated regions were identified. The *E. coli*_SAU_Sonali isolate displayed the simplest plasmid profile, carrying only an IncX1 plasmid, characterized by distinct CDS regions, GC content variation, and identifiable oriC regions, with sparse ncRNA distribution and absence of CARD‐associated genes. Comparative analysis with global *E. coli* isolates based on plasmid profile similarity revealed moderate overlap, with the highest Jaccard similarity observed for Denmark (~0.40) and the Philippines (~0.39), followed by the United States (~0.34) and Pakistan (~0.31), whereas lower similarity values were observed for countries such as India, Thailand, the United Kingdom, and Germany (~0.12–0.22) (Figure 3B). These results indicate that while Bangladeshi isolates share certain plasmid features with globally distributed strains, their overall plasmid composition remains heterogeneous, reflecting variation in plasmid content and potential region‐specific evolutionary patterns.

### 3.7 Pangenome Analysis of Poultry and Global *E. coli* Isolates

**Figure 4:**
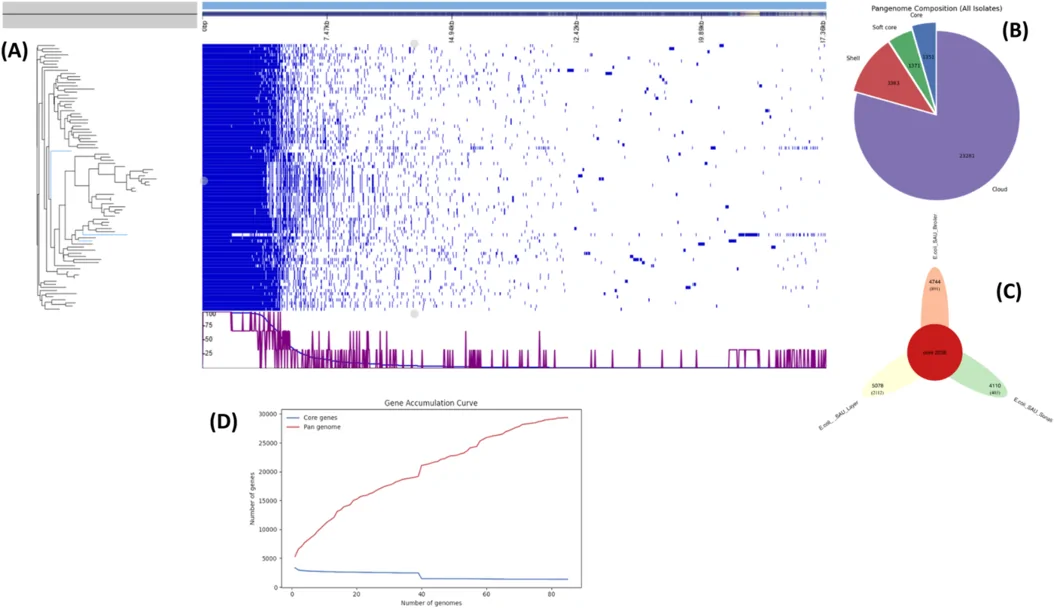
Comparative pangenome analysis of poultry and global *E. coli* isolates. (A) Gene presence–absence matrix generated from pangenome analysis, with hierarchical clustering of isolates shown on the left and gene distribution across genomes represented by colored blocks. (B) Pie chart illustrating the distribution of gene categories, including core, soft‐core, shell, and cloud genes across all analyzed genomes. (C) Flower plot depicting shared and unique gene content among the three poultry‐derived isolates, with the central region representing core genes and peripheral petals indicating isolate‐specific genes. (D) Gene accumulation curve showing the progression of core and pan‐genome size with the sequential addition of genomes.

Pangenome analysis of the three poultry‐derived *E. coli* isolates identified a total of 7550 gene clusters, of which 2238 genes (29.6%) were shared as core genes, while the remaining 5312 genes (70.4%) constituted the accessory genome. The relatively large proportion of accessory genes indicates noticeable genomic variation among the poultry isolates, which was further reflected in the flower plot (Figure 4C), where *E. coli*_SAU_Layer exhibited the highest number of unique genes (5078), followed by *E. coli*_SAU_Broiler (4744) and *E. coli*_SAU_Sonali (4110). To place these findings in a broader genomic context, the complete dataset of 86 *E. coli* genomes was analyzed, revealing a substantially larger pangenome comprising 29,366 gene clusters (Figure 4A). Among these, 1351 genes (4.6%) were identified as core, whereas the majority were distributed across soft‐core (4.7%), shell (11.4%), and cloud genes (79.3%) (Figure 4B). The gene presence–absence matrix illustrated extensive variability across isolates, which was further supported by the gene accumulation curve showing a continuous increase in total gene content with the addition of genomes (Figure 4D). While differences in gene proportions are expected due to variation in dataset size, the comparison provides a useful perspective by situating the poultry isolates within the broader genomic diversity of *E. coli*, where a relatively higher proportion of shared genes is observed among the poultry isolates compared to the more diverse global dataset. Consistent with previous studies, the low proportion of core genes (4.6%) observed across the 86 globally diverse genomes reflects the well‐established open pangenome structure of *E. coli* and is expected when genetically diverse isolates are analyzed together (Touchon et al. 2009; Rasko et al. 2008).

### 3.8 Multilocus Sequence Typing (MLST) and Clonal Complex Analysis

**Figure 5:**
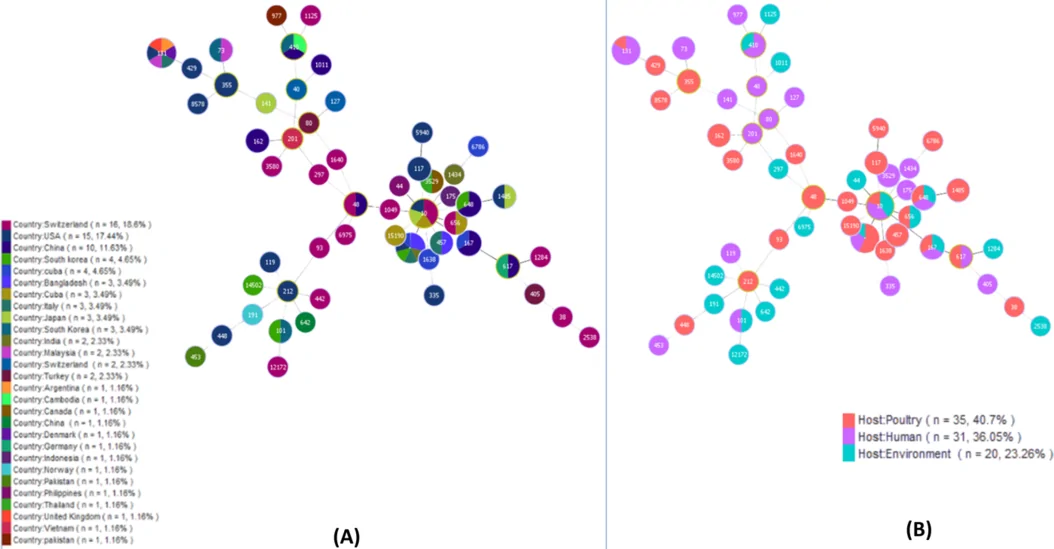
MLST‐based minimum spanning tree (MST) of *E. coli* isolates showing the global distribution of sequence types and clonal relationships. (A) MST colored by country of origin, where each node represents a sequence type (ST) and node size corresponds to the number of isolates; connecting lines indicate genetic relatedness between STs. (B) MST colored by host source, illustrating the distribution of isolates across poultry, human, and environmental origins. The Bangladeshi isolates (ST457) are positioned within a central clonal complex alongside globally distributed sequence types.

Multilocus sequence typing (MLST) analysis revealed that the three Bangladeshi *E. coli* isolates belonged to sequence type ST457, which was also identified in isolates from Italy, indicating shared sequence type membership across geographically distinct isolates. The minimum spanning tree (MST) demonstrated that ST457 clustered within a central clonal complex comprising globally distributed sequence types (Figure 5A), including ST10 (reported from Cuba, Japan, Switzerland, and the United States), ST167 (China, Cuba), ST648 (China, South Korea), and ST131 (Argentina, Denmark, Italy, Malaysia, the United Kingdom, and the United States). These sequence types were identified across multiple geographic regions, reflecting the broad global distribution of this clonal complex. The ST10 cluster appeared as a central evolutionary hub linking diverse lineages from different countries. Additional distinct clusters were observed, including an ST131‐associated group spanning multiple regions and another cluster involving ST73 (Malaysia, South Korea) and ST355 (United States), reflecting ongoing genetic diversification. Furthermore, sequence types such as ST410 (Cambodia, China, South Korea) and ST617 (China, Germany) were positioned within related subclusters, reflecting genetic relatedness and evolutionary divergence. Host distribution analysis showed that the majority of isolates originated from poultry, followed by human and environmental sources (Figure 5B), with poultry‐associated isolates contributing substantially to the central clonal complex. Overall, these findings indicate that the analyzed poultry‐derived isolates share clonal relatedness with globally distributed *E. coli* lineages.

## 4 Discussion

The present study shows that the three poultry‐derived *E. coli* isolates analyzed from Bangladesh exhibit the genomic plasticity and open pangenome architecture that have been widely reported for *E. coli*, while providing comparative genomic characterization of Bangladeshi isolates in a global context. These isolates also possess diverse antimicrobial resistance (AMR) profiles and a broad range of virulence‐associated genes. Comparative analysis further indicates that these isolates are genetically linked to globally distributed strains rather than forming a distinct regional lineage. Together, these findings suggest that the analyzed isolates may participate in broader patterns of gene exchange observed among globally distributed *E. coli* strains.

The large accessory genome observed in this study reflects the strong genomic plasticity of *E. coli*, which is largely driven by horizontal gene transfer and environmental selection pressures (Vinayamohan et al. 2022). In poultry production systems, frequent exposure to antimicrobials likely creates a selective environment that favors the retention and accumulation of resistance genes. This may explain the coexistence of multiple AMR determinants within individual isolates, as genes conferring resistance to different antibiotic classes can be co‐selected and maintained together on mobile genetic elements (Carattoli 2013). At the same time, the conserved presence of adhesion, motility, and iron acquisition genes suggests that a stable core set of virulence factors is essential for colonization and persistence in host environments (Kaper et al. 2004; Kathayat et al. 2021). In contrast, variation in secretion systems and invasion‐related genes likely reflects adaptation to specific ecological niches or host conditions. Importantly, the Jaccard similarity analysis provides additional insight into population structure. The observed moderate to high similarity with isolates from multiple countries suggests that the three Bangladeshi isolates analyzed in this study share genetic characteristics with globally distributed strains, although additional isolates are needed to determine whether this pattern is representative of the broader Bangladeshi *E. coli* population. (Abram et al. 2021; Murphy et al. 2021; Petty et al. 2014). Rather than evolving in isolation, they appear to exchange genetic material across regions, potentially through food production systems, trade networks, or environmental dissemination pathways (Murphy et al. 2021; Stoesser et al. 2017). This highlights that resistance and virulence traits are not confined to local ecosystems but circulate across geographic boundaries.

The open pangenome observed in this study is consistent with previous reports showing that *E. coli* possesses a large and continuously expanding accessory gene pool shaped by ongoing gene acquisition and loss (Touchon et al. 2009; Mcinerney et al. 2017). The continued increase in gene content with the addition of genomes reflects the well‐established open pangenome structure and extensive genomic diversity of *E. coli*, which is expected when genetically diverse isolates are analyzed together. This pattern of pangenome expansion upon the addition of new genomes underscores the remarkable genomic plasticity of the species. The antimicrobial resistance (AMR) profiles observed here closely mirror global trends reported in poultry‐associated *E. coli*, where multidrug resistance is widespread and commonly mediated by genes such as *bla*CTX‐M, *bla*TEM, *sul*, and *tet* (Van Boeckel et al. 2015; Nhung et al. 2017; Roth et al. 2016). Moreover, the frequent co‐occurrence of multiple resistance genes within individual isolates supports the well‐documented role of plasmid‐mediated horizontal gene transfer and co‐selection in driving resistance patterns (Van Boeckel et al. 2015; Carattoli 2013). Similarly, the virulence gene repertoire—particularly the conserved presence of fimbrial adhesins, iron acquisition systems, and motility genes—reflects established virulence strategies in both commensal and avian pathogenic *E. coli* (APEC) (Johnson et al. 2008; Kaper et al. 2004; Mellata 2013). Variation in accessory virulence factors, such as secretion systems and invasion‐associated genes, is consistent with previous comparative genomic studies linking such differences to strain‐specific pathogenic potential (Cordoni et al. 2016; Kathayat et al. 2021). The plasmid replicon diversity observed, dominated by IncF and Col‐type plasmids, is in line with their recognized function as major vehicles for the co‐dissemination of AMR and virulence genes (Carattoli 2013; Rozwandowicz et al. 2018). The heterogeneous plasmid content among isolates further reflects the dynamic evolution of plasmids under varying selective pressures (Smillie et al. 2010). Finally, the three Bangladeshi poultry‐derived *E. coli* isolates analyzed in this study clustered within globally distributed clonal complexes, including connections to sequence types such as ST10 and ST131, suggesting genetic relatedness to internationally reported lineages rather than a distinct regional lineage (Manges et al. 2019; Nicolas‐Chanoine et al. 2014). The detection of ST457 within this interconnected network suggests shared ancestry with internationally reported ST457 isolates rather than regional isolation; however, additional Bangladeshi isolates are required to determine whether this pattern is representative of the broader *E. coli* population. (Nesporova et al. 2021).

These findings reinforce the concept of *E. coli* as a highly adaptable organism with a flexible genome continuously shaped by gene acquisition, loss, and horizontal exchange. From a public health perspective, the presence of multidrug‐resistant and potentially virulent *E. coli* in poultry is particularly concerning, as it highlights the critical role of food‐producing animals as reservoirs of clinically relevant pathogens that can enter the food chain (Van Boeckel et al. 2015; Marshall and Levy 2011; de Mesquita Souza Saraiva et al. 2022). The observed genetic similarity between the three Bangladeshi poultry‐derived *E. coli* isolates analyzed in this study and internationally reported strains indicates shared genetic characteristics rather than direct evidence of transmission. Broader conclusions regarding Bangladesh require analysis of a larger and more geographically representative collection of isolates. (Founou et al. 2016; Mathers et al. 2015). This underscores the urgent need for strengthened antimicrobial stewardship in veterinary and agricultural sectors, improved farm‐level biosecurity measures, and integrated One Health surveillance systems that link human, animal, and environmental health to effectively limit the spread of AMR and virulence traits across interfaces (Mcewen and Collignon 2018; Robinson et al. 2016).

This study has several limitations that should be considered when interpreting the findings. The relatively small number of Bangladeshi isolates may not fully capture the diversity of *E. coli* populations in the region. In addition, the analysis is based on gene presence–absence data and does not account for gene expression or functional activity. One additional limitation relates to the assembly quality of the *E. coli*_SAU_Layer isolate. This genome exhibited substantial assembly fragmentation (6222 contigs; N50 = 1.4 kb). Reassembly of the raw sequencing reads followed by BUSCO‐based assessment yielded 62.9% complete BUSCOs, indicating that the fragmented assembly likely reflects limitations of the underlying short‐read sequencing dataset rather than assembly parameters alone. Consequently, gene prediction, pangenome estimates, and comparative genomic findings involving this isolate should be interpreted with caution. The use of publicly available global datasets may introduce sampling bias due to uneven geographic representation. Furthermore, the absence of phenotypic validation limits the ability to directly link genomic features with observable resistance or virulence traits. Furthermore, the absence of simultaneously collected poultry, human, and environmental isolates prevents inference of transmission pathways among these host compartments.

Future work should include a larger and more diverse set of isolates from different regions and production systems to better understand population‐level dynamics. Integrating transcriptomic and proteomic data would provide insight into the functional expression of identified genes. Experimental validation of key resistance and virulence determinants is also needed to confirm their biological relevance. In addition, longitudinal and One Health‐based studies incorporating human, animal, and environmental samples would help clarify transmission pathways and support the development of effective control strategies.

## 5 Conclusion

This study provides a comprehensive genomic snapshot of multidrug‐resistant *E. coli* isolated from poultry in Bangladesh. The findings indicate that the three Bangladeshi poultry‐derived *E. coli* isolates analyzed in this study exhibited the characteristic open pangenome of *E. coli* and shared genetic similarity with globally distributed strains, while providing comparative genomic insight into their antimicrobial resistance and virulence profiles. The consistent detection of ST457 within an interconnected clonal complex suggests shared ancestry with internationally reported ST457 lineages rather than direct transmission, although additional Bangladeshi isolates are needed to confirm whether this pattern is representative of the broader population. Collectively, these findings demonstrate that the analyzed poultry‐derived *E. coli* isolates harbor clinically relevant antimicrobial resistance and virulence determinants, highlighting the need for expanded genomic surveillance and antimicrobial stewardship in Bangladesh.

## Author Contributions

**Md. Rokibul Hasan Shanto:** data curation, methodology, investigation, formal analysis, validation, visualization, writing – original draft, writing – review and editing. **A. S. M. Ashab Uddin:** data curation, methodology, investigation, formal analysis, resources, visualization, writing – original draft, writing – review and editing. **Md. Shakil Mahmud Supto:** data curation, formal analysis, methodology, investigation, writing – original draft, writing – review and editing. **Nabil Jahan Mahim:** data curation, formal analysis, writing – original draft, writing – review and editing. **Md. Matiar Rahman Howlader:** methodology, supervision, writing – original draft, writing – review and editing. **Syed Sayeem Uddin Ahmed:** conceptualization, methodology, investigation, validation, formal analysis, supervision, writing – original draft, writing – review and editing. **Md Bashir Uddin:** conceptualization, methodology, investigation, validation, formal analysis, supervision, resources, project administration, visualization, writing – original draft, writing – review and editing.

## Sequence Information

The accession numbers for the *E. coli* strains included in this study‐*E. coli*_SAU_Broiler, *E. coli*_SAU_Layer, and *E. coli*_SAU_Sonali are provided in the text retrieved from NCBI. Their respective GenBank accession numbers are JBQVYW000000000, JBRNVW000000000, and JBRECO000000000.

## Conflicts of Interest

The author declare no conflicts of interest.

## Declerations

The authors declare that they have no known personal relationships that could be perceived as influencing the work reported in this paper.

## Artificial Intelligence Disclaimer

ChatGPT was used to correct grammar and improve the clarity of the manuscript text.

## Supporting information


**Figure S1: PCR amplification of the** ***E. coli.*** Representative agarose gel electrophoresis showing PCR amplification of the *16S rRNA* gene (585 bp) used for molecular confirmation of *E. coli*. M, DNA ladder; PC, positive control (*E. coli* ATCC 25922); NC, negative control; Lanes 1–9, PCR‐positive isolates

Supporting File 1

## Data Availability

All data generated or analyzed during this study are included in the article or in the Supporting files.
