# Supplementary material for: Comparative Genomic Analysis of Multidrug‐Resistant Escherichia coli Across Poultry–Human–Environmental Interfaces
Source: Microbiologyopen. 2026 Sep 13;15(5):e70406. doi: 10.1002/mbo3.70406 (PMC13572854; doi:10.1002/mbo3.70406)
Supplement: Supplementary file 1 [file MBO3-15-e70406-s002.docx]

**Supplementary Table S1. Metadata and source information of E. coli isolates included in this study.**

| **Accession ID** | **Biosample** | **Strain** | **Host** | **Isolation Source** | **Country** | **year** |
| --- | --- | --- | --- | --- | --- | --- |
| [GCA_052492195.1 (*E. coli*_SAU_Broiler)](https://www.ncbi.nlm.nih.gov/datasets/genome/GCF_052492195.1/) | [SAMN50861382](https://www.ncbi.nlm.nih.gov/biosample/SAMN50861382/) | ARAC-SAU-CH-3615 | Poultry | Feces | Bangladesh | 2020 |
| [GCA_053051155.1 (E. coli_SAU_Layer)](https://www.ncbi.nlm.nih.gov/datasets/genome/GCA_053051155.1/) | [SAMN51409036](https://www.ncbi.nlm.nih.gov/biosample/SAMN51409036/) | ARAC-SAU-CH-3762 | Poultry | Feces | Bangladesh | 2020 |
| [GCA_052906445.1 (E. coli_SAU_Sonali)](https://www.ncbi.nlm.nih.gov/datasets/genome/GCF_052906445.1/) | [SAMN51409148](https://www.ncbi.nlm.nih.gov/biosample/SAMN51409148/) | ARAC-SAU-CH-3688 | Poultry | Feces | Bangladesh | 2020 |
| [GCA_055382605.1](https://www.ncbi.nlm.nih.gov/datasets/genome/GCA_055382605.1/) | [SAMD00874696](https://www.ncbi.nlm.nih.gov/biosample/SAMD00874696) | BroCaecum-55 | Poultry | Feces | Japan | 2024 |
| [GCA_024225435.1](https://www.ncbi.nlm.nih.gov/datasets/genome/GCA_024225435.1/) | [SAMN29499313](https://www.ncbi.nlm.nih.gov/biosample/SAMN29499313) | LH13-b | Poultry | Feces | Switzerland | 2022 |
| [GCA_025369775.1](https://www.ncbi.nlm.nih.gov/datasets/genome/GCA_025369775.1/) | [SAMN30789308](https://www.ncbi.nlm.nih.gov/biosample/SAMN30789308) | E5 | Poultry | - | USA | 2017 |
| [GCA_025369795.1](https://www.ncbi.nlm.nih.gov/datasets/genome/GCA_025369795.1/) | [SAMN30789310](https://www.ncbi.nlm.nih.gov/biosample/SAMN30789310) | E7 | Poultry | - | USA | 2017 |
| [GCA_025369815.1](https://www.ncbi.nlm.nih.gov/datasets/genome/GCA_025369815.1/) | [SAMN30789312](https://www.ncbi.nlm.nih.gov/biosample/SAMN30789312) | E9 | Poultry | - | USA | 2017 |
| [GCA_001660565.1](https://www.ncbi.nlm.nih.gov/datasets/genome/GCA_001660565.1/) | [SAMN05190012](https://www.ncbi.nlm.nih.gov/biosample/SAMN05190012) | S51 | Poultry | Meat | Switzerland | 2015 |
| [GCA_024223415.1](https://www.ncbi.nlm.nih.gov/datasets/genome/GCA_024223415.1/) | [SAMN29499317](https://www.ncbi.nlm.nih.gov/biosample/SAMN29499317) | LH50-c | Poultry | Feces | Switzerland | 2022 |
| [GCA_026506355.1](https://www.ncbi.nlm.nih.gov/datasets/genome/GCA_026506355.1/) | [SAMN28550360](https://www.ncbi.nlm.nih.gov/biosample/SAMN28550360) | MS1679 | Poultry | Lung | USA | 2022 |
| [GCA_033098385.1](https://www.ncbi.nlm.nih.gov/datasets/genome/GCA_033098385.1/) | [SAMN37560772](https://www.ncbi.nlm.nih.gov/biosample/SAMN37560772) | MS1657 | Poultry | Heart | USA | 2020 |
| [GCA_026506315.1](https://www.ncbi.nlm.nih.gov/datasets/genome/GCA_026506315.1/) | [SAMN28550362](https://www.ncbi.nlm.nih.gov/biosample/SAMN28550362) | MS1737 | Poultry | Blood | USA | 2021 |
| [GCA_026506335.1](https://www.ncbi.nlm.nih.gov/datasets/genome/GCA_026506335.1/) | [SAMN28550361](https://www.ncbi.nlm.nih.gov/biosample/SAMN28550361) | MS1718 | Poultry | Blood | USA | 2021 |
| [GCA_025369695.1](https://www.ncbi.nlm.nih.gov/datasets/genome/GCA_025369695.1/) | [SAMN30789311](https://www.ncbi.nlm.nih.gov/biosample/SAMN30789311) | E8 | Poultry | - | USA | 2017 |
| [GCA_024224055.1](https://www.ncbi.nlm.nih.gov/datasets/genome/GCA_024224055.1/) | [SAMN29499321](https://www.ncbi.nlm.nih.gov/biosample/SAMN29499321) | LH85-a | Poultry | Feces | Switzerland | 2022 |
| [GCA_033098885.1](https://www.ncbi.nlm.nih.gov/datasets/genome/GCA_033098885.1/) | [SAMN37560771](https://www.ncbi.nlm.nih.gov/biosample/SAMN37560771) | MS1266 | Poultry | Spleen | USA | 2019 |
| [GCA_024585465.1](https://www.ncbi.nlm.nih.gov/datasets/genome/GCA_024585465.1/) | [SAMN18877315](https://www.ncbi.nlm.nih.gov/biosample/SAMN18877315) | MB53 | Poultry | Cloaca | Cuba | 2014 |
| [GCA_053323875.1](https://www.ncbi.nlm.nih.gov/datasets/genome/GCA_053323875.1/) | [SAMN52951588](https://www.ncbi.nlm.nih.gov/biosample/SAMN52951588) | E3 | Poultry | - | China | 2024 |
| [GCA_024585505.1](https://www.ncbi.nlm.nih.gov/datasets/genome/GCA_024585505.1/) | [SAMN18877313](https://www.ncbi.nlm.nih.gov/biosample/SAMN18877313) | MB51 | Poultry | Cloaca | Cuba | 2014 |
| [GCA_024585425.1](https://www.ncbi.nlm.nih.gov/datasets/genome/GCA_024585425.1/) | [SAMN18877317](https://www.ncbi.nlm.nih.gov/biosample/SAMN18877317) | MB57 | Poultry | Cloaca | Cuba | 2014 |
| [GCA_042138005.1](https://www.ncbi.nlm.nih.gov/datasets/genome/GCA_042138005.1/) | [SAMN43472425](https://www.ncbi.nlm.nih.gov/biosample/SAMN43472425) | AH50 | Poultry | Liver | China | 2012 |
| [GCA_025583295.2](https://www.ncbi.nlm.nih.gov/datasets/genome/GCA_025583295.2/) | [SAMN30724613](https://www.ncbi.nlm.nih.gov/biosample/SAMN30724613) | YZMc17-1 | Poultry | Feces | China | 2019 |
| [GCA_041897545.1](https://www.ncbi.nlm.nih.gov/datasets/genome/GCA_041897545.1/) | [SAMN43473463](https://www.ncbi.nlm.nih.gov/biosample/SAMN43473463) | EC052 | Poultry | Liver | China | 2022 |
| [GCA_024225755.1](https://www.ncbi.nlm.nih.gov/datasets/genome/GCA_024225755.1/) | [SAMN29499312](https://www.ncbi.nlm.nih.gov/biosample/SAMN29499312) | LH10-b-1 | Poultry | Feces | Switzerland | 2022 |
| [GCA_024585585.1](https://www.ncbi.nlm.nih.gov/datasets/genome/GCA_024585585.1/) | [SAMN18877309](https://www.ncbi.nlm.nih.gov/biosample/SAMN18877309) | MB31 | Poultry | Cloaca | cuba | 2015 |
| [GCA_024585605.1](https://www.ncbi.nlm.nih.gov/datasets/genome/GCA_024585605.1/) | [SAMN18877308](https://www.ncbi.nlm.nih.gov/biosample/SAMN18877308) | MB30 | Poultry | Cloaca | cuba | 2015 |
| [GCA_025583375.2](https://www.ncbi.nlm.nih.gov/datasets/genome/GCA_025583375.2/) | [SAMN30724602](https://www.ncbi.nlm.nih.gov/biosample/SAMN30724602) | YZLc23-1 | Poultry | Feces | China | 2019 |
| [GCA_024226085.1](https://www.ncbi.nlm.nih.gov/datasets/genome/GCA_024226085.1/) | [SAMN29499310](https://www.ncbi.nlm.nih.gov/biosample/SAMN29499310) | LH09-a | Poultry | Feces | Switzerland | 2022 |
| [GCA_016767775.1](https://www.ncbi.nlm.nih.gov/datasets/genome/GCA_016767775.1/) | [SAMN14503865](https://www.ncbi.nlm.nih.gov/biosample/SAMN14503865) | MS1170 | Poultry | yolk sac | USA | 2019 |
| [GCA_024585485.1](https://www.ncbi.nlm.nih.gov/datasets/genome/GCA_024585485.1/) | [SAMN18877314](https://www.ncbi.nlm.nih.gov/biosample/SAMN18877314) | MB52 | Poultry | Cloaca | cuba | 2014 |
| [GCA_024585645.1](https://www.ncbi.nlm.nih.gov/datasets/genome/GCA_024585645.1/) | [SAMN18877306](https://www.ncbi.nlm.nih.gov/biosample/SAMN18877306) | MB20 | Poultry | Cloaca | cuba | 2015 |
| [GCA_026506375.1](https://www.ncbi.nlm.nih.gov/datasets/genome/GCA_026506375.1/) | [SAMN28550359](https://www.ncbi.nlm.nih.gov/biosample/SAMN28550359) | MS1665 | Poultry | Heart | USA | 2020 |
| [GCA_039725815.1](https://www.ncbi.nlm.nih.gov/datasets/genome/GCA_039725815.1/) | [SAMN16872742](https://www.ncbi.nlm.nih.gov/biosample/SAMN16872742) | EC-10 | Poultry | Cloaca | Italy | 2017 |
| [GCA_041021985.1](https://www.ncbi.nlm.nih.gov/datasets/genome/GCA_041021985.1/) | [SAMN41822573](https://www.ncbi.nlm.nih.gov/biosample/SAMN41822573) | 723_010814_2_1 | Poultry | Salpingitis | Denmark | 2014 |
| [GCA_055380745.1](https://www.ncbi.nlm.nih.gov/datasets/genome/GCA_055380745.1/) | [SAMD01599414](https://www.ncbi.nlm.nih.gov/biosample/SAMD01599414) | AST46 | Human | Stool | Japan | 2002 |
| [GCA_055378535.1](https://www.ncbi.nlm.nih.gov/datasets/genome/GCA_055378535.1/) | [SAMD01599420](https://www.ncbi.nlm.nih.gov/biosample/SAMD01599420) | AST216 | Human | Stool | Japan | 2020 |
| [GCA_001617565.1](https://www.ncbi.nlm.nih.gov/datasets/genome/GCA_001617565.1/) | [SAMN04621897](https://www.ncbi.nlm.nih.gov/biosample/SAMN04621897) | Ecol_732 | Human | - | Thailand | 2012 |
| [GCA_001566615.1](https://www.ncbi.nlm.nih.gov/datasets/genome/GCA_001566615.1/) | [SAMN04381849](https://www.ncbi.nlm.nih.gov/biosample/SAMN04381849) | SaT040 | Human | - | USA | 2007 |
| [GCA_001644745.1](https://www.ncbi.nlm.nih.gov/datasets/genome/GCA_001644745.1/) | [SAMN04851091](https://www.ncbi.nlm.nih.gov/biosample/SAMN04851091) | 2013C-4465 | Human | Stool | USA | 2013 |
| [GCA_001618365.1](https://www.ncbi.nlm.nih.gov/datasets/genome/GCA_001618365.1/) | [SAMN04590097](https://www.ncbi.nlm.nih.gov/biosample/SAMN04590097) | Ecol_448 | Human | - | Argentina | 2011 |
| [GCA_001577325.1](https://www.ncbi.nlm.nih.gov/datasets/genome/GCA_001577325.1/) | [SAMN04381853](https://www.ncbi.nlm.nih.gov/biosample/SAMN04381853) | ZH063 | Human | - | Canada | 2002 |
| [GCA_001420935.1](https://www.ncbi.nlm.nih.gov/datasets/genome/GCA_001420935.1/) | [SAMN03952643](https://www.ncbi.nlm.nih.gov/biosample/SAMN03952643) | 2012C-4227 | Human | - | USA | 2012 |
| [GCA_001043215.1](https://www.ncbi.nlm.nih.gov/datasets/genome/GCA_001043215.1/) | [SAMN03659038](https://www.ncbi.nlm.nih.gov/biosample/SAMN03659038) | NCM3722 | Human | - | USA | 2014 |
| [GCA_002879735.1](https://www.ncbi.nlm.nih.gov/datasets/genome/GCA_002879735.1/) | [SAMN08242382](https://www.ncbi.nlm.nih.gov/biosample/SAMN08242382) | Ec40 | Human | Stool | India | 2013 |
| [GCA_040105125.1](https://www.ncbi.nlm.nih.gov/datasets/genome/GCA_040105125.1/) | [SAMN41610980](https://www.ncbi.nlm.nih.gov/biosample/SAMN41610980) | ST-131 | Human | Urine | India | 2017 |
| [GCA_003028775.1](https://www.ncbi.nlm.nih.gov/datasets/genome/GCA_003028775.1/) | [SAMN07196154](https://www.ncbi.nlm.nih.gov/biosample/SAMN07196154) | E24377A | Human | Urine | Pakistan | 2016 |
| [GCA_052907575.1](https://www.ncbi.nlm.nih.gov/datasets/genome/GCA_052907575.1/) | [SAMN51205882](https://www.ncbi.nlm.nih.gov/biosample/SAMN51205882) | 700462 | Human | Stool | pakistan | 2008 |
| [GCA_001860505.1](https://www.ncbi.nlm.nih.gov/datasets/genome/GCA_001860505.1/) | [SAMN04299569](https://www.ncbi.nlm.nih.gov/biosample/SAMN04299569) | Y5 | Human | Urine | China | 2013 |
| [GCA_001485455.1](https://www.ncbi.nlm.nih.gov/datasets/genome/GCA_001485455.1/) | [SAMN02800875](https://www.ncbi.nlm.nih.gov/biosample/SAMN02800875) | ST648 | Human | Pleural Effusion | China | 2009 |
| [GCA_055362145.1](https://www.ncbi.nlm.nih.gov/datasets/genome/GCA_055362145.1/) | [SAMN50743354](https://www.ncbi.nlm.nih.gov/biosample/SAMN50743354) | ESBLEC_HM24 | Human | Rectal swab | Malaysia | 2024 |
| [GCA_053850535.1](https://www.ncbi.nlm.nih.gov/datasets/genome/GCA_053850535.1/) | [SAMN50743356](https://www.ncbi.nlm.nih.gov/biosample/SAMN50743356) | ESBLEC_HM52 | Human | Rectal swab | Malaysia | 2024 |
| [GCA_001683435.1](https://www.ncbi.nlm.nih.gov/datasets/genome/GCA_001683435.1/) | [SAMN05291917](https://www.ncbi.nlm.nih.gov/biosample/SAMN05291917) | K-15KW01 | Human | - | Switzerland | 2015 |
| [GCA_008370755.1](https://www.ncbi.nlm.nih.gov/datasets/genome/GCA_008370755.1/) | [SAMN12275742](https://www.ncbi.nlm.nih.gov/biosample/SAMN12275742) | ESBL 15 | Human | Rectum | Switzerland | 2013 |
| [GCA_002012085.1](https://www.ncbi.nlm.nih.gov/datasets/genome/GCA_002012085.1/) | [SAMN05511152](https://www.ncbi.nlm.nih.gov/biosample/SAMN05511152) | Ecol_AZ146 | Human | - | Italy | 2012 |
| [GCA_040267545.1](https://www.ncbi.nlm.nih.gov/datasets/genome/GCA_040267545.1/) | [SAMN40468454](https://www.ncbi.nlm.nih.gov/biosample/SAMN40468454) | TA_NDM-5 | Human | Rectal swab | Italy | 2023 |
| [GCA_044647535.1](https://www.ncbi.nlm.nih.gov/datasets/genome/GCA_044647535.1/) | [SAMN44321997](https://www.ncbi.nlm.nih.gov/biosample/SAMN44321997) | HS-01 | Human | Stool | Indonesia | 2024 |
| [GCA_002787935.1](https://www.ncbi.nlm.nih.gov/datasets/genome/GCA_002787935.1/) | [SAMN07944241](https://www.ncbi.nlm.nih.gov/biosample/SAMN07944241) | CREC-591 | Human | Peritoneal fluid | South Korea | 2015 |
| [GCA_001693315.1](https://www.ncbi.nlm.nih.gov/datasets/genome/GCA_001693315.1/) | [SAMN05392487](https://www.ncbi.nlm.nih.gov/biosample/SAMN05392487) | UPEC 26-1 | Human | Urine | South Korea | 2012 |
| [GCA_002953455.1](https://www.ncbi.nlm.nih.gov/datasets/genome/GCA_002953455.1/) | [SAMN08369073](https://www.ncbi.nlm.nih.gov/biosample/SAMN08369073) | KBN10P04869 | Human | Blood | South Korea | 2017 |
| [GCA_011403355.1](https://www.ncbi.nlm.nih.gov/datasets/genome/GCA_011403355.1/) | [SAMD00190256](https://www.ncbi.nlm.nih.gov/biosample/SAMD00190256) | 2017.15.01CC | Human | - | Vietnam | 2017 |
| [GCA_006536085.3](https://www.ncbi.nlm.nih.gov/datasets/genome/GCA_006536085.3/) | [SAMD00164387](https://www.ncbi.nlm.nih.gov/biosample/SAMD00164387) | NIPH17_0020 | Human | - | Cambodia | 2017 |
| [GCA_014898615.1](https://www.ncbi.nlm.nih.gov/datasets/genome/GCA_014898615.1/) | [SAMN16330218](https://www.ncbi.nlm.nih.gov/biosample/SAMN16330218) | 188B | Human | Blood | Turkey | 2018 |
| [GCA_050897925.1](https://www.ncbi.nlm.nih.gov/datasets/genome/GCA_050897925.1/) | [SAMN48819743](https://www.ncbi.nlm.nih.gov/biosample/SAMN48819743) | NA22 | Human | Blood | Turkey | 2025 |
| [GCA_001886535.1](https://www.ncbi.nlm.nih.gov/datasets/genome/GCA_001886535.1/) | [SAMN06029893](https://www.ncbi.nlm.nih.gov/biosample/SAMN06029893) | MRSN352231 | Human | Clinical | Germany | 2016 |
| [GCA_001469815.1](https://www.ncbi.nlm.nih.gov/datasets/genome/GCA_001469815.1/) | [SAMN04159541](https://www.ncbi.nlm.nih.gov/biosample/SAMN04159541) | uk_P46212 | Human | Urine | United Kingdom | 2005 |
| [GCA_008642415.1](https://www.ncbi.nlm.nih.gov/datasets/genome/GCA_008642415.1/) | [SAMN10784802](https://www.ncbi.nlm.nih.gov/biosample/SAMN10784802) | 1500 | Environment | Marine environment | Norway | 2014 |
| [GCA_055807955.1](https://www.ncbi.nlm.nih.gov/datasets/genome/GCA_055807955.1/) | [SAMN55925752](https://www.ncbi.nlm.nih.gov/biosample/SAMN55925752) | 33E | Environment | Farm | Switzerland | 2023 |
| [GCA_030384685.1](https://www.ncbi.nlm.nih.gov/datasets/genome/GCA_030384685.1/) | [SAMN35722724](https://www.ncbi.nlm.nih.gov/biosample/SAMN35722724) | VG1B2 | Environment | Farm | Switzerland | 2021 |
| [GCA_053668395.1](https://www.ncbi.nlm.nih.gov/datasets/genome/GCA_053668395.1/) | [SAMN53269467](https://www.ncbi.nlm.nih.gov/biosample/SAMN53269467) | L16-131 | Environment | Lake | Philippines | 2024 |
| [GCA_039654195.1](https://www.ncbi.nlm.nih.gov/datasets/genome/GCA_039654195.1/) | [SAMN41243834](https://www.ncbi.nlm.nih.gov/biosample/SAMN41243834) | WWCOL-315 | Environment | Waste water | South korea | 2024 |
| [GCA_030384805.1](https://www.ncbi.nlm.nih.gov/datasets/genome/GCA_030384805.1/) | [SAMN35722718](https://www.ncbi.nlm.nih.gov/biosample/SAMN35722718) | VG35A2 | Environment | Farm | Switzerland | 2021 |
| [GCA_030384765.1](https://www.ncbi.nlm.nih.gov/datasets/genome/GCA_030384765.1/) | [SAMN35722720](https://www.ncbi.nlm.nih.gov/biosample/SAMN35722720) | VG48B2 | Environment | Farm | Switzerland | 2021 |
| [GCA_030169635.1](https://www.ncbi.nlm.nih.gov/datasets/genome/GCA_030169635.1/) | [SAMN30667245](https://www.ncbi.nlm.nih.gov/biosample/SAMN30667245) | AHM21C2872I | Environment | In-house | China | 2021 |
| [GCA_055807935.1](https://www.ncbi.nlm.nih.gov/datasets/genome/GCA_055807935.1/) | [SAMN55925753](https://www.ncbi.nlm.nih.gov/biosample/SAMN55925753) | 45E | Environment | Farm | Switzerland | 2022 |
| [GCA_031884895.1](https://www.ncbi.nlm.nih.gov/datasets/genome/GCA_031884895.1/) | [SAMN37422107](https://www.ncbi.nlm.nih.gov/biosample/SAMN37422107) | E7-1N7 | Environment | Agricultural environment | South korea | 2022 |
| [GCA_027944995.1](https://www.ncbi.nlm.nih.gov/datasets/genome/GCA_027944995.1/) | [SAMN31988404](https://www.ncbi.nlm.nih.gov/biosample/SAMN31988404) | DETEC-E223 | Environment | - | China | 2019 |
| [GCA_010365425.1](https://www.ncbi.nlm.nih.gov/datasets/genome/GCA_010365425.1/) | [SAMN13951922](https://www.ncbi.nlm.nih.gov/biosample/SAMN13951922) | 10 | Environment | - | Switzerland | 2019 |
| [GCA_047300905.1](https://www.ncbi.nlm.nih.gov/datasets/genome/GCA_047300905.1/) | [SAMN46224883](https://www.ncbi.nlm.nih.gov/biosample/SAMN46224883) | WWCOL-236 | Environment | Waste water | South korea | 2023 |
| [GCA_030384825.1](https://www.ncbi.nlm.nih.gov/datasets/genome/GCA_030384825.1/) | [SAMN35722717](https://www.ncbi.nlm.nih.gov/biosample/SAMN35722717) | VG12A2 | Environment | Farm | Switzerland | 2021 |
| [GCA_029909715.1](https://www.ncbi.nlm.nih.gov/datasets/genome/GCA_029909715.1/) | [SAMN30916723](https://www.ncbi.nlm.nih.gov/biosample/SAMN30916723) | GYX208DH4E-2 | Environment | - | China | 2020 |
| [GCA_023612455.1](https://www.ncbi.nlm.nih.gov/datasets/genome/GCA_023612455.1/) | [SAMN18062621](https://www.ncbi.nlm.nih.gov/biosample/SAMN18062621) | MEI003 | Environment | Outdoors environment of a veterinary clinic | Switzerland | 2019 |
| [GCA_047300885.1](https://www.ncbi.nlm.nih.gov/datasets/genome/GCA_047300885.1/) | [SAMN46224884](https://www.ncbi.nlm.nih.gov/biosample/SAMN46224884) | WWCOL-256 | Environment | Waste water | South korea | 2023 |
| [GCA_023612415.1](https://www.ncbi.nlm.nih.gov/datasets/genome/GCA_023612415.1/) | [SAMN18062623](https://www.ncbi.nlm.nih.gov/biosample/SAMN18062623) | MEI005 | Environment | Outdoors environment of a veterinary clinic | Switzerland | 2019 |
| [GCA_010365345.1](https://www.ncbi.nlm.nih.gov/datasets/genome/GCA_010365345.1/) | [SAMN13951925](https://www.ncbi.nlm.nih.gov/biosample/SAMN13951925) | 32-4 | Environment | - | Switzerland | 2019 |
| [GCA_020034635.1](https://www.ncbi.nlm.nih.gov/datasets/genome/GCA_020034635.1/) | [SAMN20959237](https://www.ncbi.nlm.nih.gov/biosample/SAMN20959237) | JNU | Environment | Water | China | 2006 |

**Supplementary Table S2: Antimicrobial susceptibility with 95% Confidence Interval (n = 280)**

| **Antibiotic** | **Sensitive,**  **n (%)** | **95% CI** | **Resistant,**  **n (%)** | **95% CI** |
| --- | --- | --- | --- | --- |
| Ampicillin (AMP) | 6 (2.14) | 0.99–4.60 | 274 (97.86) | 95.40–99.01 |
| Tetracycline (TET) | 7 (2.50) | 1.22–5.07 | 273 (97.50) | 94.93–98.78 |
| Amoxicillin-clavulanate (AMC) | 244 (87.14) | 82.71–90.57 | 36 (12.86) | 9.43–17.29 |
| Ciprofloxacin (CIP) | 87 (31.07) | 25.94–36.72 | 193 (68.93) | 63.28–74.06 |
| Gentamicin (CN) | 186 (66.43) | 60.71–71.70 | 94 (33.57) | 28.30–39.29 |
| Sulfamethoxazole-trimethoprim (SXT) | 23 (8.21) | 5.54–12.02 | 257 (91.79) | 87.98–94.46 |
| Azithromycin (AZM) | 270 (96.43) | 93.55–98.05 | 10 (3.57) | 1.95–6.45 |
| Imipenem (IMP) | 270 (96.43) | 93.55–98.05 | 10 (3.57) | 1.95–6.45 |
| Ceftriaxone (CRO) | 219 (78.21) | 73.02–82.65 | 61 (21.79) | 17.35–26.98 |
| Ceftazidime (CAZ) | 242 (86.43) | 81.92–89.95 | 38 (13.57) | 10.05–18.08 |
| Cefoxitin (FOX) | 129 (46.07) | 40.33–51.92 | 151 (53.93) | 48.08–59.67 |
| Cefepime (FEP) | 135 (48.21) | 42.43–54.05 | 145 (51.79) | 45.95–57.57 |
